# Potential of circulating pro‐angiogenic microRNA expressions as biomarkers for rapid angiographic stenotic progression and restenosis risks in coronary artery disease patients underwent percutaneous coronary intervention

**DOI:** 10.1002/jcla.23013

**Published:** 2019-09-08

**Authors:** Rui Dai, Yijue Liu, Yi Zhou, Xiaoju Xiong, Wei Zhou, Weijuan Li, Wenping Zhou, Manhua Chen

**Affiliations:** ^1^ Department of Cardiology, Tongji Medical College The Central Hospital of Wuhan, Huazhong University of Science & Technology Wuhan China; ^2^ Emergency Department, Tongji Medical College The Central Hospital of Wuhan, Huazhong University of Science & Technology Wuhan China

**Keywords:** coronary artery disease, percutaneous coronary intervention, pro‐angiogenesis microRNA, rapid angiographic stenotic progression, restenosis

## Abstract

**Background:**

This study aimed to investigate the correlation of pro‐angiogenic microRNA (miRNA) expressions with rapid angiographic stenotic progression (RASP) and restenosis risks in coronary artery disease (CAD) patients underwent percutaneous coronary intervention (PCI) with drug‐eluting stents (DES).

**Methods:**

A total of 286 CAD patients underwent PCI with DES were consecutively recruited in this study. Plasma samples were collected before PCI operation, and 14 pro‐angiogenic miRNAs were measured by real‐time quantitative reverse transcription‐polymerase chain reaction. Rapid angiographic stenotic progression at nontarget lesions and restenosis at stented lesions were evaluated by quantitative coronary angiography at 12 months after PCI operation.

**Results:**

The occurrence rates of RASP and restenosis were 39.5% and 22.4%, respectively. Let‐7f, miR‐19a, miR‐19b‐1, miR‐92a, miR‐126, miR‐210, and miR‐296 were decreased in RASP patients than non‐RASP patients, among which let‐7f, miR‐19a, miR‐126, miR‐210, and miR‐296 independently correlated with lower RASP occurrence by multivariate analysis, followed by receiver‐operating characteristic (ROC) curve exhibited that these five miRNAs showed great value in predicting RASP risk with area under curve (AUC) 0.879 (95% CI: 0.841‐0.917). Besides, let‐7f, miR‐19a, miR‐92a, miR‐126, miR‐130a, and miR‐210 were reduced in restenosis patients than non‐restenosis patients, among them miR‐19a, miR‐126, miR‐210, and miR‐378 independently correlated with lower restenosis occurrence by multivariate analysis, followed by ROC curve disclosed that these four miRNAs had good value in predicting restenosis risk with AUC 0.776 (95% CI: 0.722‐0.831).

**Conclusions:**

Circulating let‐7f, miR‐19a, miR‐126, miR‐210, and miR‐296 independently correlate with reduced RASP risk, while miR‐19a, miR‐126, miR‐210, and miR‐378 independently correlate with decreased restenosis risk in CAD patients underwent PCI with DES.

## 1 INTRODUCTION

Coronary artery disease (CAD) is the leading cause of deaths in both developing and developed countries with the annual deaths more than 17.5 million worldwide, accounting for approximately one third of all‐cause deaths.^1^, ^2^, ^3^ In clinical practice, there are several revascularization treatments for CAD, such as percutaneous coronary intervention (PCI), coronary artery bypass graft surgery (CABG), and drug therapy, among which PCI represents the most common therapy for CAD patients, and PCI with drug‐eluting stents (DES) achieves significant relief of clinical symptoms and reduction in mortality in CAD patients compared with previous bare‐metal stents (BMS).^4^, ^5^, ^6^, ^7^, ^8^, ^9^ However, in clinical practice, CAD patients underwent PCI with DES may also present with rapid angiographic stenotic progression (RASP) at nontarget lesion and restenosis at stented lesion in CAD patients. Although antiproliferative drugs loaded in stents are designed to reduce the occurrence of RASP as well as restenosis which have made some progresses, especially restenosis rate could decrease to 3%‐20%, while the incidences of RASP and restenosis are still not negligible due to a very large CAD population treated by PCI with DES.^10^, ^11^, ^12^, ^13^ Hence, it is meaningful to explore better strategies for predicting RASP and restenosis risks in CAD patients underwent PCI with DES, thereby guiding optimization of clinical treatment strategies and taking preventive measures as soon as possible.

Angiogenesis is of great importance in the development and progression of CAD by expanding microvascular network and assisting vascular repair to improve vascular functions.^14^ Accordingly, pro‐angiogenic microRNA (miRNA) is a class of approximately 20‐25 nt, single‐stranded noncoding RNAs that regulate vascular functions through post‐transcriptional modulation of anti‐angiogenic genes expressions, and a few pro‐angiogenic miRNAs have been identified until now.^15^, ^16^, ^17^ For instance, miR‐130a induces angiogenesis through targeting C‐MYB in vascular endothelial cells^15^; miR‐126 and miR‐210 improve angiogenesis and then present with cardioprotective effect in rat.^17^ Since angiogenesis is benefiting for the microvascular network rebuilding and vascular repair, we hypothesized that pro‐angiogenic miRNA expressions might correlate with the risks of RASP and restenosis in CAD patients underwent PCI with DES, while no related data have been reported. Therefore, we selected 14 pro‐angiogenic miRNAs referring to a previous comprehensive review on angiogenesis‐related miRNAs,^18^ and this study aimed to explore the correlation of these 14 pro‐angiogenic miRNA expressions with RASP and restenosis risks in CAD patients underwent PCI with DES.

## 2 METHODS

### 2.1 Participants

During the period from January 2012 to December 2016, 286 CAD patients who underwent PCI with DES implantation at the Central Hospital of Wuhan were consecutively recruited in this study. The inclusion criteria included (a) confirmed diagnosis of severe CAD based on the coronary angiography, which was defined as the luminal stenosis ≥70% in at least one major coronary vessel, (b) age ≥18 years old, (c) scheduled to undergo PCI with DES implantation, and (d) able to be regularly followed up. Patients were excluded if they (a) had contraindications to the PCI, (b) were allergic to the medications: clopidogrel, rapamycin, paclitaxel, stainless steel, or cobalt alloy, (c) previously underwent major surgery, PCI, or coronary artery bypass grafting, (d) received treatment with immunosuppressive drugs within 3 months, (e) complicated with inflammatory diseases, autoimmune diseases, (f) with a history of severe infection or malignancies, and (g) were pregnant women or lactating women. The present study was approved by the Institutional Review Board of the Central Hospital of Wuhan and conducted in accordance with the Declaration of Helsinki. All patients provided the written informed consents before enrollment.

### 2.2 Data collection

After the eligibility was confirmed and the informed consents were provided, patients' baseline features were recorded, which included age, gender, body mass index (BMI), smoke, hypertension, diabetes mellitus, hypercholesteremia, hyperuricemia, and family history of CAD and left ventricular ejection fraction (LVEF), and the laboratory detections were also recorded including mean arterial pressure (MAP), fasting blood glucose (FBG), serum creatinine (Scr), serum uric acid (SUA), cardiac troponin I (cTnI), N‐terminal pro‐brain natriuretic peptide (NT‐proBNP), triglyceride (TG), total cholesterol (TC), high‐density lipoprotein cholesterol (HDL‐C), low‐density lipoprotein cholesterol (LDL‐C), high‐sensitivity C‐reactive protein (Hs‐CRP), erythrocyte sedimentation rate (ESR), white blood cell (WBC), and neutrophil. Moreover, characteristics of lesions were collected after coronary angiography, such as multivessel artery lesions, target lesion at left anterior descending branch (LAD), target lesion at left circumflex artery (LCX), target lesion at right coronary artery (RCA), percentage of patients with two target lesions, stenosis degree of target lesion, and length of target lesion. And the parameters of PCI surgery (such as length of stent, diameter of stent, time of stent dilation, and balloon dilation pre‐stent) as well as drugs used after surgery (such as aspirin, nitrates, statins, β receptor blockers, angiotensin‐converting enzyme inhibitors/angiotensin receptor blockers (ACEIs/ARBs), and calcium channel blockers) were recorded accordingly.

### 2.3 Measurement of candidate miRNAs

Blood samples were collected from all patients before PCI and centrifuged at 880 *g* for 10 minutes to separate plasma. Total RNA was extracted from the plasma using TRIzol solution (Invitrogen) according to the manufacturer's protocol. The extracted RNA in solution was stored at −80°C until use. RNA concentration and purity from all samples were measured using NanoDrop ND‐1000 Spectrophotometer (Thermo Fisher Scientific). The integrity of RNA was determined by 1.2% agarose electrophoresis (Sigma). Before real‐time quantitative reverse transcription‐polymerase chain reaction (RT‐qPCR) was performed, the complementary DNA (cDNA) was synthesized from total RNA using a RevertAid RT Reverse Transcription Kit (Thermo Fisher Scientific) following the manufacturer's instructions. RT‐qPCR was carried out on an Applied Biosystems 7900 HT thermocycler (Applied Biosystems) with TB Green™ Fast qPCR Mix (Takara), and the reactions were incubated at 95°C for 3 minutes, followed by 40 cycles of 95°C for 5 seconds and 61°C for 30 seconds. All reactions were run in triplicate. Relative quantitation of gene expression was analyzed by the 2^−ΔΔCq^ method, and the U6 was utilized as an internal reference for normalizing miRNA quantity. The primers used in the study are listed in Table S1.

### 2.4 Quantitative coronary angiography (QCA) analysis

The procedures of PCI and DES implantation and the perioperative managements were performed in accordance with the recommendations of the guidelines of PCI, and the rapamycin‐eluting stent and paclitaxel‐eluting stent were used as DES (the choice of stent depended on the patients' willingness). Quantitative coronary angiography was performed before PCI (baseline), post‐operation, and at 12th month after PCI or earlier if indicated by clinical symptoms or evidence of myocardial ischemia. Coronary angiograms were obtained after intracoronary nitroglycerin administration. Baseline, postoperative, and follow‐up coronary angiograms were digitally recorded, and the QCA analyses were performed with an automated edge‐detection system (Medis Medical Imaging Systems) by experienced specialists.

### 2.5 Definitions

Rapid angiographic stenotic progression of nontarget lesion and restenosis of stented lesions were evaluated by QCA. Rapid angiographic stenotic progression of nontarget lesion was defined as the occurrence of any of the following conditions^19^: (a) the increase in stenosis ≥10% at 12th month if the original stenosis was ≥50% before PCI; (b) the increase in stenosis ≥30% at 12th month if the original stenosis was <50% before PCI; (c) newly developed stenosis ≥30% at 12th month if no original stenosis existed before PCI; (d) and the stenosis aggravated and turned to complete occlusion lesion at 12th month. Restenosis of stented lesion was defined as the lumen stenosis at stent‐implanted segment was ≥50% at 12th month (compared with postoperative).

### 2.6 Statistics analysis

Statistical analysis was performed with the use of SPSS 22.0 statistical software (SPSS Inc), and figures were made using GraphPad Prism 7.00 software (GraphPad Software Inc). Data were expressed as count (percentage), mean ± standard deviation, or median (25th‐75th quantiles). Comparison was determined by the Wilcoxon rank‐sum test. Univariate and multivariate logistic regression model analyses with the forward stepwise (conditional) method were used to determine miRNAs related to the RASP or restenosis occurrence. Receiver‐operating characteristic (ROC) curves and the area under the ROC curve (AUC) were used to assess the predictive value of miRNAs for the RASP or restenosis occurrence. *P* value < .05 was considered significant.

## 3 RESULTS

### 3.1 Study flow

**Figure 1:**
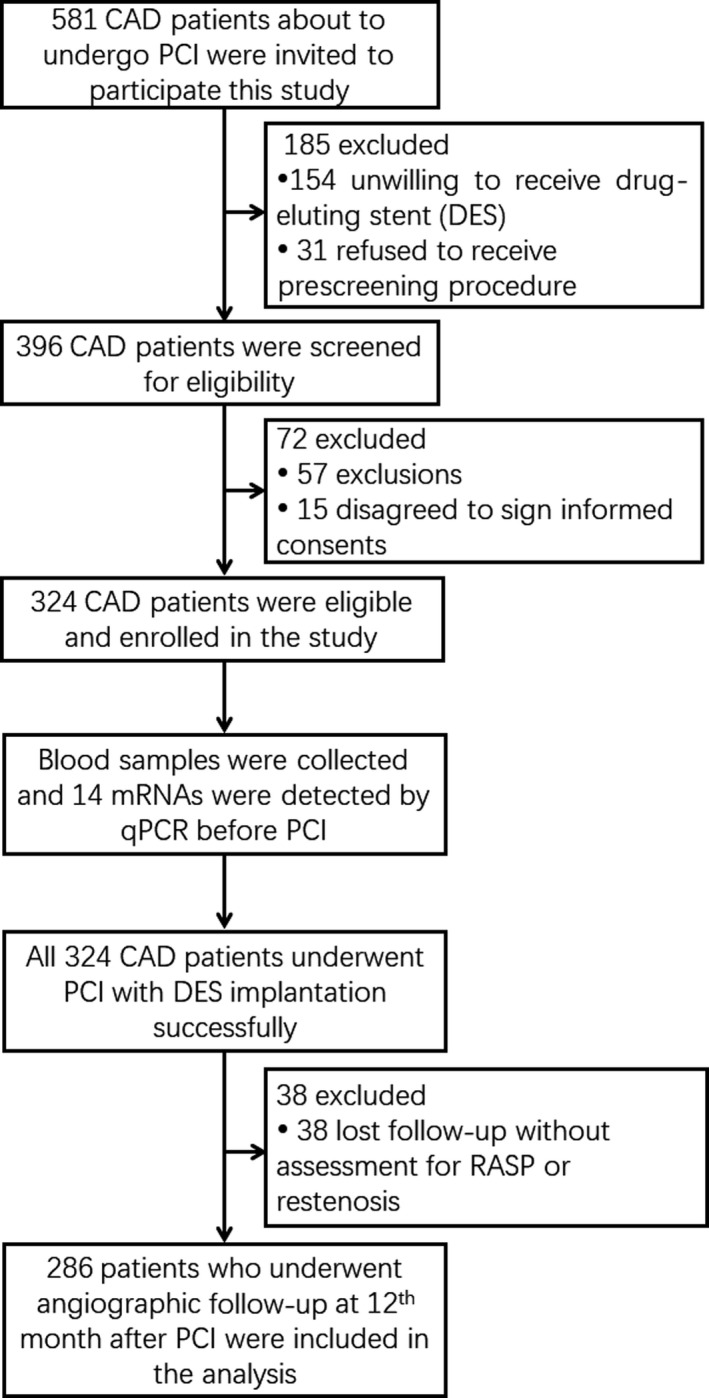
Study flow

Initially, 581 CAD patients who were about to undergo PCI were invited to participate in this study, while 185 patients were excluded, among which 154 patients were unwilling to receive DES and 31 patients refused to receive prescreening procedure. The left 396 CAD patients were screened for eligibility, and 72 of them were excluded, among which 57 patients disobeyed inclusion criteria or met exclusion criteria and 15 patients disagreed to sign informed consents. The remaining 324 CAD patients were eligible and enrolled in the study. Before PCI, plasma samples from all enrolled patients were collected and 14 mRNAs in plasma were detected by RT‐qPCR. All 324 CAD patients successfully underwent PCI with DES implantation, among which 38 patients were excluded from final analysis due to losing follow‐up without assessment for RASP or restenosis. Finally, 286 patients were included in the analysis (Figure 1).

### 3.2 Baseline characteristics

The mean age of CAD patients was 60.4 ± 9.6 years with mean BMI 25.9 ± 3.4 kg/m^2^, and 232 (81.1%) males as well as 54 (18.9%) females were included (Table S2). There were 216 (75.5%), 165 (57.7%), 103 (36.0%), 102 (35.7%), and 84 (29.4%) patients with multivessel artery lesions, target lesion at LAD, target lesion at LCX, target lesion at RCA, and two target lesions, respectively. Additionally, the median stenosis degree of target lesion was 88.0% (84.0%‐92.0%), and the median length of target lesion was 34.0 (27.0‐41.0) mm. The other detailed baseline characteristics, including cardiovascular risk factors, laboratory detections, parameters of PCI with DES, and medicine used post‐operation, are listed in Table S2.

### 3.3 The occurrence rates of RASP and restenosis

**Figure 2:**
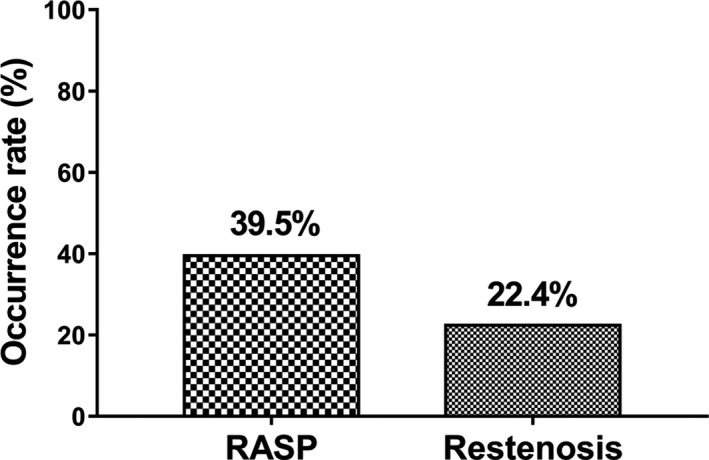
RASP and restenosis occurrence. The occurrence rate of RASP and restenosis was 39.5% and 22.4%, respectively. RASP, rapid angiographic stenotic progression

There were 113 cases (39.5%) and 64 cases (22.4%) occurred RASP and restenosis, respectively, in CAD patients underwent PCI with DES implantation (Figure 2).

### 3.4 Comparison of pro‐angiogenic miRNA expression levels between RASP and non‐RASP patients

**Figure 3:**
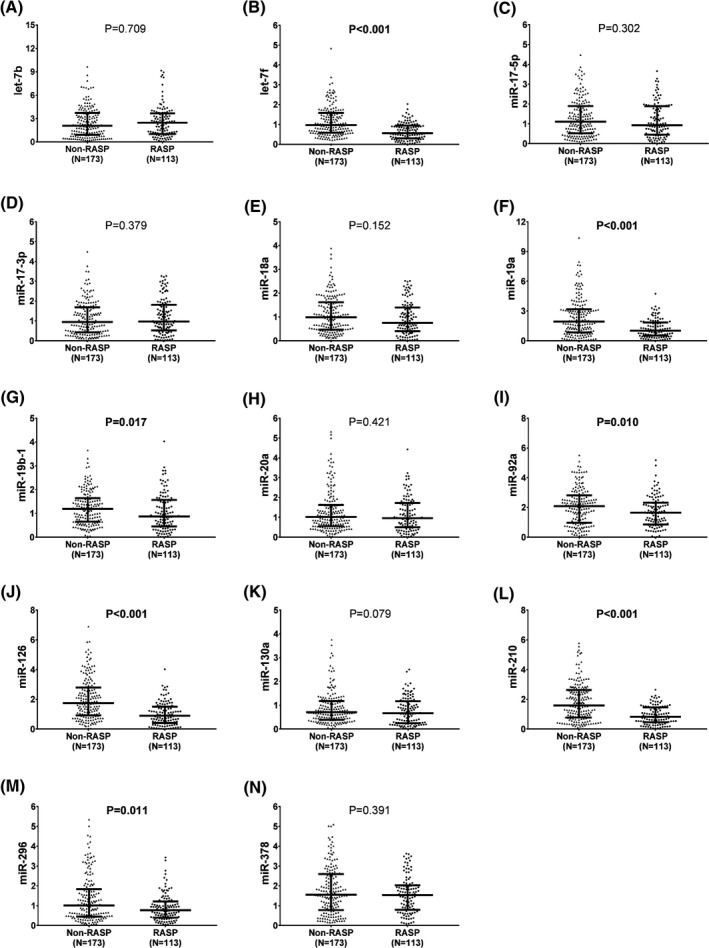
Pro‐angiogenic miRNA expression levels in RASP and non‐RASP patients. Lower baseline expression levels of let‐7f (B), miR‐19a (F), miR‐19b‐1 (G), miR‐92a (I), miR‐126 (J), miR‐210 (L), and miR‐296 (M) were observed in RASP patients compared with non‐RASP patients, while no difference in the other seven miRNA expression levels (including let‐7b (A), miR‐17‐5p (C), miR‐17‐3p (D), miR‐18a (E), miR‐20a (H), miR‐130a (K), and miR‐378 (N)) at baseline was discovered between RASP patients and non‐RASP patients. Comparison was determined by the Wilcoxon rank‐sum test. *P* < .05 was considered as significant. miRNA, microRNA; RASP, rapid angiographic stenotic progression

Compared to non‐RASP patients, RASP patients exhibited lower expression levels of let‐7f (*P* < .001) (Figure 3B), miR‐19a (*P* < .001) (Figure 3F), miR‐19b‐1 (*P* = .017) (Figure 3G), miR‐92a (*P* = .010) (Figure 3I), miR‐126 (*P* < .001) (Figure 3J), miR‐210 (*P* < .001) (Figure 3L), and miR‐296 (*P* = .011) (Figure 3M) at baseline, while there was no difference in the other seven candidate miRNA expression levels between non‐RASP and RASP patients at baseline, including let‐7b (Figure 3A), miR‐17‐5p (Figure 3C), miR‐17‐3p (Figure 3D), miR‐18a (Figure 3E), miR‐20a (Figure 3H), miR‐130a (Figure 3K), and miR‐378 (Figure 3N).

### 3.5 Correlation of pro‐angiogenic miRNA expressions with RASP risk

In order to further investigate the correlation of pro‐angiogenic miRNA expressions with RASP risk, univariate and multivariate logistic regression analyses were performed. Univariate logistic regression analysis displayed that let‐7f (*P* < .001, OR: 0.216, 95% CI: 0.127‐0.368), miR‐19a (*P* < .001, OR: 0.575, 95% CI: 0.465‐0.711), miR‐92a (*P* = .008, OR: 0.745, 95% CI: 0.599‐0.926), miR‐126 (*P* < .001, OR: 0.409, 95% CI: 0.305‐0.549), miR‐210 (*P* < .001, OR: 0.374, 95% CI: 0.271‐0.517), and miR‐296 (*P* = .001, OR: 0.613, 95% CI: 0.462‐0.812) were correlated with lower RASP risk (Table S3). Further multivariate logistic regression analysis revealed that let‐7f (*P* < .001, OR: 0.293, 95% CI: 0.155‐0.554), miR‐19a (*P* < .001, OR: 0.571, 95% CI: 0.435‐0.750), miR‐126 (*P* < .001, OR: 0.433, 95% CI: 0.306‐0.614), miR‐210 (*P* < .001, OR: 0.404, 95% CI: 0.271‐0.604), and miR‐296 (*P* = .027, OR: 0.645, 95% CI: 0.437‐0.952) were independently correlated with decreased RASP risk.

### 3.6 Predictive value of pro‐angiogenic miRNAs for RASP risk determined by ROC curves

**Figure 4:**
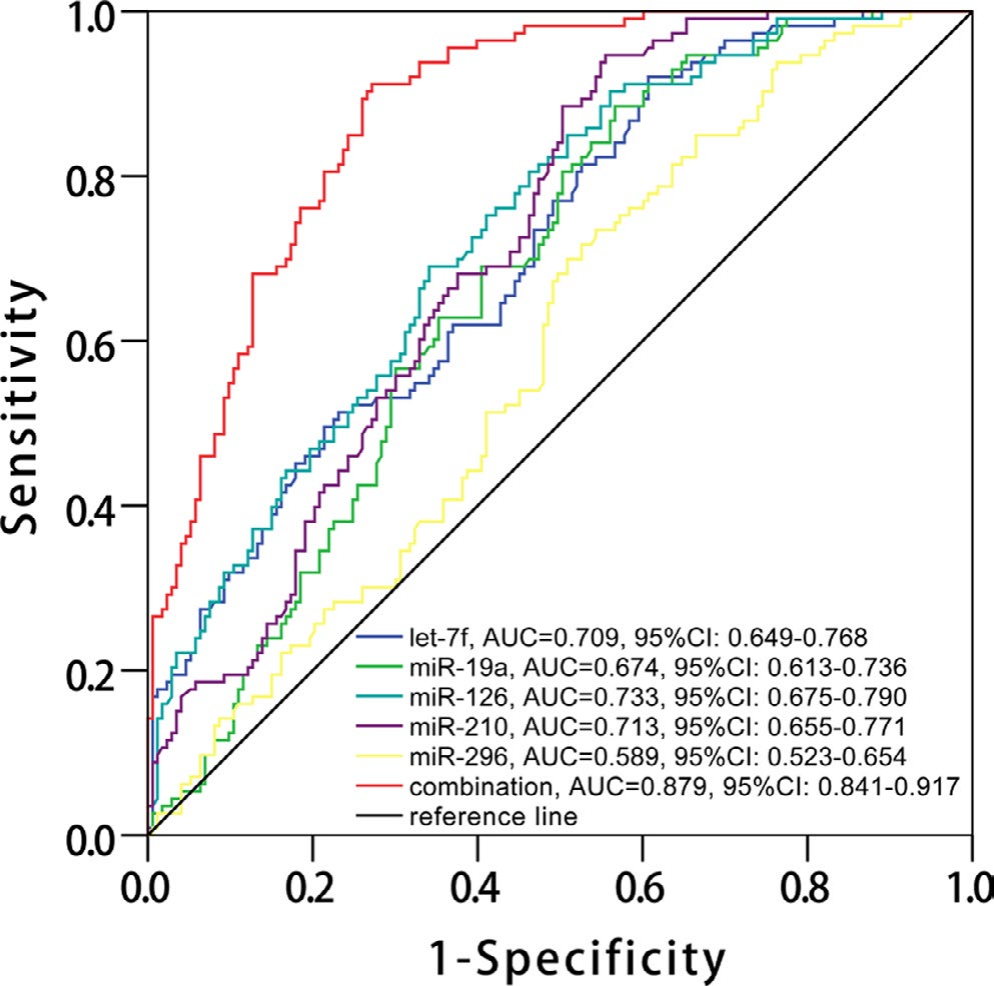
ROC curve analysis for RASP risk. ROC curve disclosed that let‐7f, miR‐19a, miR‐126, miR‐210, and miR‐296 could predict RASP risk, and combination of all these five miRNAs presented even better predictive value for RASP occurrence (red color line). The analysis was determined by ROC curve analysis. RASP, rapid angiographic stenotic progression; ROC, receiver‐operating characteristic

Since let‐7f, miR‐19a, miR‐126, miR‐210, and miR‐296 independently correlated with decreased RASP occurrence, ROC curve analysis was further carried out to evaluate their predictive value for RASP risk, which disclosed that let‐7f (AUC: 0.709, 95% CI: 0.649‐0.768), miR‐19a (AUC: 0.674, 95% CI: 0.613‐0.736), miR‐126 (AUC: 0.733, 95% CI: 0.675‐0.790), miR‐210 (AUC: 0.713, 95% CI: 0.655‐0.771), and miR‐296 (AUC: 0.589, 95% CI: 0.523‐0.654) could predict RASP risk (Figure 4). Moreover, combination of all these five miRNAs displayed even better predictive value for RASP risk with AUC 0.879 (95% CI: 0.841‐0.917).

### 3.7 Comparison of pro‐angiogenic miRNA expression levels between restenosis and non‐restenosis patients

**Figure 5:**
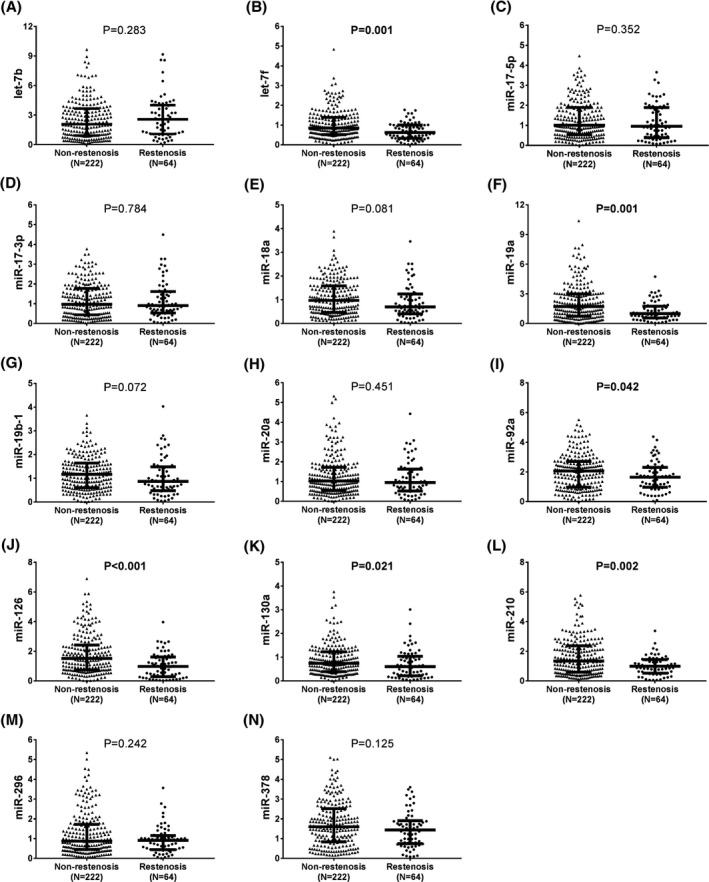
Pro‐angiogenic miRNA expression levels in restenosis and non‐restenosis patients. Decreased baseline expression levels of let‐7f (B), miR‐19a (F), miR‐92a (I), miR‐126 (J), miR‐130a (K), and miR‐210 (L) were observed in restenosis patients compared with non‐restenosis patients, while no difference in the other eight miRNA expression levels (including let‐7b (A), miR‐17‐5p (C), miR‐17‐3p (D), miR‐18a (E), miR‐19b‐1 (G), miR‐20a (H), miR‐296 (M), and miR‐378 (N)) at baseline was discovered between restenosis patients and non‐restenosis patients. Comparison was determined by the Wilcoxon rank‐sum test. *P* < .05 was considered as significant. miRNA, microRNA

Compared to non‐restenosis patients, restenosis patients displayed decreased expression levels of let‐7f (*P* = .001) (Figure 5B), miR‐19a (*P* < .001) (Figure 5F), miR‐92a (*P* = .042) (Figure 5I), miR‐126 (*P* < .001) (Figure 5J), miR‐130a (*P* = .021) (Figure 5K), and miR‐210 (*P* = .002) (Figure 5L) at baseline, whereas the other candidate eight miRNA expression levels did not differ between non‐restenosis and restenosis patients at baseline, including let‐7b (Figure 3A), miR‐17‐5p (Figure 3C), miR‐17‐3p (Figure 3D), miR‐18a (Figure 3E), miR‐19b‐1 (Figure 3G), miR‐20a (Figure 3H), miR‐296 (Figure 3M), and miR‐378 (Figure 3N).

### 3.8 Correlation of pro‐angiogenic miRNA expressions with restenosis risk

To assess the correlation of pro‐angiogenic expressions with restenosis risk, univariate and multivariate logistic regression analyses were carried out. Univariate logistic regression analysis uncovered that let‐7f (*P* = .001, OR: 0.376, 95% CI: 0.214‐0.660), miR‐19a (*P* < .001, OR: 0.641, 95% CI: 0.500‐0.820), miR‐92a (*P* = .042, OR: 0.765, 95% CI: 0.591‐0.991), miR‐126 (*P* < .001, OR: 0.510, 95% CI: 0.369‐0.706), miR‐210 (*P* < .001, OR: 0.540, 95% CI: 0.385‐0.757), and miR‐296 (*P* = .041, OR: 0.710, 95% CI: 0.511‐0.986) were associated with reduced restenosis risk (Table 1). Further multivariate logistic regression analysis disclosed that miR‐19a (*P* = .001, OR: 0.645, 95% CI: 0.495‐0.841), miR‐126 (*P* < .001, OR: 0.530, 95% CI: 0.376‐0.746), miR‐210 (*P* = .009, OR: 0.610, 95% CI: 0.421‐0.885), and miR‐378 (*P* = .029, OR: 0.712, 95% CI: 0.525‐0.965) were independently associated with lower restenosis risk.

**Table 1:** Predictive effect of miRNA relative expression on restenosis by logistic regression analysis

miRNAs	Logistic regression model			
	*P* value	OR	95% CI	
			Lower	Higher
Univariate logistic regression				
let‐7b	.290	1.077	0.939	1.235
let‐7f	**.001**	0.376	0.214	0.660
miR‐17‐5p	.429	0.881	0.643	1.207
miR‐17‐3p	.972	0.994	0.723	1.367
miR‐18a	.093	0.706	0.471	1.059
miR‐19a	**<.001**	0.641	0.500	0.820
miR‐19b‐1	.185	0.765	0.515	1.137
miR‐20a	.389	0.876	0.649	1.183
miR‐92a	**.042**	0.765	0.591	0.991
miR‐126	**<.001**	0.510	0.369	0.706
miR‐130a	.066	0.625	0.378	1.032
miR‐210	**<.001**	0.540	0.385	0.757
miR‐296	**.041**	0.710	0.511	0.986
miR‐378	.070	0.776	0.589	1.021
Multivariate logistic regression with forward stepwise (conditional) method				
miR‐19a	**.001**	0.645	0.495	0.841
miR‐126	**<.001**	0.530	0.376	0.746
miR‐210	**.009**	0.610	0.421	0.885
miR‐378	**.029**	0.712	0.525	0.965

Note

Predictive effect of miRNA relative expression on restenosis was determined by univariate and multivariate logistic regression analyses, and the multivariate logistic regression analysis was performed with forward stepwise (conditional) method. *P* value < .05 was considered significant (in bold).

Abbreviations: CI, confidence interval; OR, odds ratio.

### 3.9 Predictive value of pro‐angiogenic miRNAs for restenosis risk determined by ROC curves

**Figure 6:**
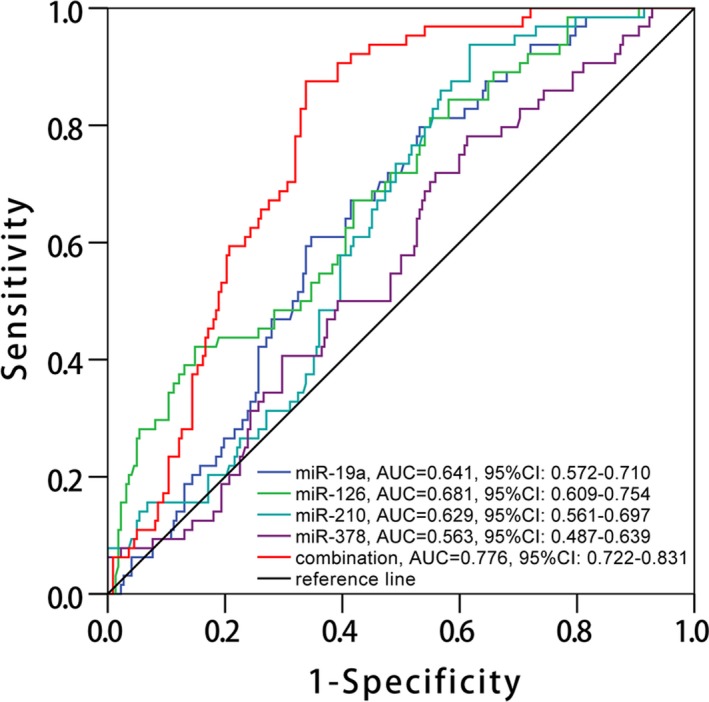
ROC curve analysis for restenosis risk. ROC curve revealed that miR‐19a, miR‐126, and miR‐210 could predict restenosis risk, while miR‐378 could not, and combination of all these four miRNAs displayed good predictive value for restenosis occurrence (red color line). The analysis was determined by ROC curve analysis. miRNA, microRNA; ROC, receiver‐operating characteristic

Since miR‐19a, miR‐126, miR‐210, and miR‐378 independently correlated with decreased restenosis occurrence, ROC curve analysis was further carried out to evaluate their value for predicting restenosis risk, which revealed that miR‐19a (AUC: 0.641, 95% CI: 0.572‐0.710), miR‐126 (AUC: 0.681, 95% CI: 0.609‐0.754), and miR‐210 (AUC: 0.629, 95% CI: 0.561‐0.697) could predict restenosis risk, while miR‐378 could not (AUC: 0.563, 95% CI: 0.487‐0.639) (Figure 6). Furthermore, combination of all these four miRNAs exhibited higher predictive value for restenosis risk with AUC 0.776 (95% CI: 0.722‐0.831).

## 4 DISCUSSION

In the present study**,** we observed that in CAD patients underwent PCI with DES: (a) circulating let‐7f, miR‐19a, miR‐126, miR‐210, and miR‐296 independently correlated with reduced RASP risk, and their combination presented with great predictive value for RASP risk with AUC 0.879 (95% CI: 0.841‐0.917); and (b) circulating miR‐19a, miR‐126, miR‐210, and miR‐378 independently associated with decreased restenosis risk, and their combination illuminated good predictive value for restenosis risk with AUC 0.776 (95% CI: 0.722‐0.831).

Rapid angiographic stenotic progression at non‐stented sites and restenosis at stented sites are two common major risks in CAD patients underwent PCI with DES. Rapid angiographic stenotic progression is characterized by a rapid plaque progression and a consequent luminal obstruction at nontarget locations, which possibly arises from plaques disruption and endothelial mechanical injury caused by stent implantation lead to accretion of exfoliated plaques, endothelial dysfunction, and subsequent responsive processes including inflammatory reaction, ectopic proliferation, and migration of vascular smooth muscle cells (VSMCs) at nontarget sites.^20^, ^21^, ^22^ As to restenosis, it occurs at stented sites probability due to the vulnerability of stented arteries and the effect of regeneration of endothelium, which manifests as distinct neointimal hyperplasia at stented sites.^21^, ^22^ Briefly, RASP and restenosis derive from several adverse effects caused by stent implantation, including endothelial cell (EC) dysfunction, ectopic proliferation, and migration of VSMCs as well as inflammatory reaction.^3^, ^22^

Recently, accumulating studies disclose that pro‐angiogenic miRNAs play critical roles in vascular remodeling and pathophysiology of cardiovascular diseases.^23^, ^24^, ^25^, ^26^, ^27^, ^28^, ^29^, ^30^, ^31^, ^32^, ^33^, ^34^, ^35^ For instance, miR‐126, as a common reported pro‐angiogenesis gene, promotes vessel wound healing and attenuates development of cardiovascular pathology by inducing angiogenesis and vascular regeneration in vascular injury and ischemic conditions,^23^, ^24^ and improves angiogenic function of ECs or endothelial progenitor cells (EPCs) via regulating epidermal growth factor‐like domain 7 (EGFL7)^25^; meanwhile, it reduces ectopic VSMC proliferation and limits neointima formation by inhibiting low‐density lipoprotein receptor‐related protein 6 (LRP6).^28^ In addition, miR‐126 is also a negative regulator of inflammation, which is a key contributor to cardiovascular pathology, including endothelial dysfunction and ectopic proliferation and migration of VSMCs.^26^, ^27^, ^28^ As to another common pro‐angiogenic miRNA, miR‐92a facilitates angiogenesis via upregulating vascular endothelial growth factor A (VEGFA) and re‐endothelialization, preventing neointima formation following vascular injury,^30^, ^31^ and it protects VSMCs against apoptosis via suppressing of both mitogen‐activated protein kinase 4 (MKK4) and c‐Jun N‐terminal kinase (JNK) pathways in vascular remodeling of several vascular diseases, such as aneurysms, post‐angioplasty restenosis, and atherosclerosis, which leads to inhibition of plaque instability and rupture.^32^ Furthermore, another pro‐angiogenic miR‐210 is also observed to accelerate angiogenesis via activating notch signaling pathway and able to decrease inflammatory cells density as well as reduce the apoptosis and dysfunction of ECs through inhibiting the receptor tyrosine kinase ligand Ephrin‐A3, protecting from vascular ischemic damage.^33^, ^34^, ^35^ Considering the functions of pro‐angiogenesis miRNAs in vascular remodeling and the pathological processes of RASP and restenosis in blood vessels, we hypothesized that pro‐angiogenic miRNAs might contribute to less progression (including less occurrences of RASP and restenosis) in CAD patients underwent PCI with DES implantation; thus, we conducted this present study to verify this assumption.

It has been recently disclosed that several pro‐angiogenic miRNAs are applied as novel biomarkers for susceptibility and progression of several vascular diseases in clinical practice.^36^, ^37^, ^38^ For example, a study reveals that plasma pro‐angiogenic miR‐126, miR‐130a, miR‐222, miR‐218, and miR‐185 are identified as independent predictive factors for reduced acute ischemic stroke (AIS) risk, and miR‐126, miR‐378, miR‐101, miR‐222, miR‐218, and miR‐206 are associated with AIS progression assessed by National Institutes of Health Stroke Scale score.^38^ Another study illuminates that peripheral blood miR‐210 correlates with decreased risk of chronic obstructive pulmonary disease combined with ischemic stroke.^39^ Besides, a recent study also observes that miR‐126, miR‐17‐5p, miR‐92a, miR‐210, and miR‐378 independently correlate with reduced risk of CAD and disease progression (assessed by Gensini score).^40^ These data provide evidences for the potential of pro‐angiogenic miRNAs to serve as biomarkers for risk and progression of vascular diseases, while no report has been exhibited about their correlations with RASP and restenosis risk in CAD patients underwent PCI with DES implantation. In this study, we discovered that circulating let‐7f, miR‐19a, miR‐126, miR‐210, and miR‐296 independently correlated with reduced RASP risk, and combination of their combination presented with great predictive value for RASP risk with AUC 0.879, while circulating miR‐19a, miR‐126, miR‐210, and miR‐378 independently associated with decreased restenosis risk, and their combination illuminated good predictive value for restenosis risk with 0.776 in CAD patients underwent PCI with DES implantation. The possible explanations of these results might be that (a) these pro‐angiogenic miRNAs could induce angiogenesis, vascular repair, and re‐endothelialization through activating ECs (or EPCs) and suppressing their apoptosis or inhibits ectopic proliferation and apoptosis of VSMCs, thereby obstruct plaque rupture and neointima formation following vascular injury at nontarget locations and stented sites. (b) Besides, these pro‐angiogenic miRNAs might suppress inflammatory reactions via reducing related protein expressions, such as mitogen‐activated protein kinase phosphatase 1 (MKP‐1) and vascular cell adhesion molecule‐1 (VCAM‐1), leading to amelioration of endothelial dysfunction and suppression of ectopic VSMC proliferation.^41^, ^42^

There were several limitations in this study. Firstly, angiographic surveillance of RASP and restenosis might elevate the restenosis rate; hence, other alternatives of assessment could be utilized, such as intravascular ultrasonography, optical coherence tomography, and fractional flow reserve.^43^ Secondly, we only assessed the short‐term incidence of RASP or restenosis in CAD patients underwent PCI with DES; thereby, further study would be performed with longer follow‐up time. Thirdly, this was a single‐center study with relatively low patients which might lead to patient selection bias and reduced statistical efficiency; thus, further study with more medical centers and patients should be conducted in the future.

In conclusion, circulating let‐7f, miR‐19a, miR‐126, miR‐210, and miR‐296 independently correlate with reduced RASP risk, while miR‐19a, miR‐126, miR‐210, and miR‐378 independently correlate with decreased restenosis risk in CAD patients underwent PCI with DES.

## CONFLICT OF INTEREST

The authors declare that they have no conflicts of interest.
