# Supplementary material for: Potential of circulating pro‐angiogenic microRNA expressions as biomarkers for rapid angiographic stenotic progression and restenosis risks in coronary artery disease patients underwent percutaneous coronary intervention
Source: J Clin Lab Anal. 2019 Sep 8;34(1):e23013. doi: 10.1002/jcla.23013 (PMC6977144; doi:10.1002/jcla.23013)
Supplement: Supplementary file 1 [file JCLA-34-e23013-s001.docx]

**SUPPLEMENTARY TABLE 1** Primers used in the study

| Gene | Forward (5'->3') | Reverse (5'->3') |
| --- | --- | --- |
| let-7b | ACACTCCAGCTGGGTGAGGTAGTAGGTTGTGT | ACACTCCAGCTGGGACTGCAGTGAAGGCACTT |
| let-7f | ACACTCCAGCTGGGTGAGGTAGTAGATTGTAT | ACACTCCAGCTGGGACTGCAGTGAAGGCACTT |
| miR-17-5p | ACACTCCAGCTGGGTGAGGTAGTAGGTTGTGT | ACACTCCAGCTGGGACTGCAGTGAAGGCACTT |
| miR-17-3p | ACACTCCAGCTGGGACTGCAGTGAAGGCACTT | ACACTCCAGCTGGGACTGCAGTGAAGGCACTT |
| miR-18a | ACACTCCAGCTGGGTAAGGTGCATCTAGTGCA | ACACTCCAGCTGGGACTGCAGTGAAGGCACTT |
| miR-19a | ACACTCCAGCTGGGAGTTTTGCATAGTTGCAC | ACACTCCAGCTGGGACTGCAGTGAAGGCACTT |
| miR-19b-1 | ACACTCCAGCTGGGAGTTTTGCAGGTTTGCAT | ACACTCCAGCTGGGACTGCAGTGAAGGCACTT |
| miR-20a | ACACTCCAGCTGGGTAAAGTGCTTATAGTGCA | ACACTCCAGCTGGGACTGCAGTGAAGGCACTT |
| miR-92a | ACACTCCAGCTGGGTATTGCACTTGTCCCGGC | ACACTCCAGCTGGGACTGCAGTGAAGGCACTT |
| miR-126 | ACACTCCAGCTGGGCATTATTACTTTTGGTAC | ACACTCCAGCTGGGACTGCAGTGAAGGCACTT |
| miR-130a | ACACTCCAGCTGGGTTCACATTGTGCTACTGT | ACACTCCAGCTGGGACTGCAGTGAAGGCACTT |
| miR-210 | ACACTCCAGCTGGGAGCCCCTGCCCACCGCAC | ACACTCCAGCTGGGACTGCAGTGAAGGCACTT |
| miR-296 | ACACTCCAGCTGGGAGGGCCCCCCCTCAATCC | ACACTCCAGCTGGGACTGCAGTGAAGGCACTT |
| miR-378 | ACACTCCAGCTGGGCTCCTGACTCCAGGTCCT | ACACTCCAGCTGGGACTGCAGTGAAGGCACTT |
| U6 | CTCGCTTCGGCAGCACA | AACGCTTCACGAATTTGCGT |
