# Supplementary material for: Potential of circulating pro‐angiogenic microRNA expressions as biomarkers for rapid angiographic stenotic progression and restenosis risks in coronary artery disease patients underwent percutaneous coronary intervention
Source: J Clin Lab Anal. 2019 Sep 8;34(1):e23013. doi: 10.1002/jcla.23013 (PMC6977144; doi:10.1002/jcla.23013)
Supplement: Supplementary file 2 [file JCLA-34-e23013-s002.docx]

**SUPPLEMENTARY TABLE 2** Characteristics of CAD patients

| Items | CAD patents (N=286) |
| --- | --- |
| **Demographic features** |  |
| Age (years) | 60.4 ± 9.6 |
| Gender |  |
| Male (n/%) | 232 (81.1) |
| Female (n/%) | 54 (18.9) |
| BMI (kg/m^2^) | 25.9 ± 3.4 |
| **Cardiovascular risk factors** |  |
| Smoke (n/%) | 98 (34.3) |
| Hypertension (n/%) | 217 (75.9) |
| DM (n/%) | 80 (28.0) |
| Hypercholesteremia (n/%) | 169 (59.1) |
| Hyperuricemia (n/%) | 118 (41.3) |
| Family history of CAD (n/%) | 48 (16.8) |
| LVEF (%) | 65.0 (61.0-70.0) |
| **Laboratory detections** |  |
| MAP (mmHg) | 103.95 ± 17.68 |
| FBG (mmol/L) | 5.73 (5.11-6.56) |
| Glycated hemoglobin (%) | 6.00 (4.90-7.30) |
| Scr (μmol/L) | 80.34 ± 16.54 |
| SUA (μmol/L) | 339.97 ± 81.93 |
| cTnI (ng/mL) | 0.029 (0.017-0.042) |
| NT-proBNP (ng/mL) | 0.077 (0.041-0.117) |
| TG (mmol/L) | 1.74 (1.00-2.46) |
| TC (mmol/L) | 4.65 ± 1.00 |
| LDL-C (mmol/L) | 2.80 ± 0.64 |
| HDL-C (mmol/L) | 1.02 (0.83-1.19) |
| Hs-CRP (mg/L) | 5.81 (2.30-10.27) |
| ESR (mm/L) | 16.30 (8.92-24.34) |
| WBC (x10^9^/L) | 6.01 (4.85-7.08) |
| Neutrophil (x10^9^/L) | 3.49 ± 0.98 |
| **Characteristics of lesions** |  |
| Multivessel artery lesions (n/%) | 216 (75.5) |
| Target lesion at LAD (n/%) | 165 (57.7) |
| Target lesion at LCX (n/%) | 103 (36.0) |
| Target lesion at RCA (n/%) | 102 (35.7) |
| Patients with two target lesions (n/%) | 84 (29.4) |
| Stenosis degree of target lesion (%) | 88.0 (84.0-92.0) |
| Length of target lesion (mm) | 34.0 (27.0-41.0) |
| **Parameters of PCI with DES** |  |
| Bifurcation requiring double wiring (n/%) | 83 (29.0) |
| Length of stent (mm) | 38.0 (31.0-44.0) |
| Diameter of stent (mm) | 3.2 (3.0-3.4) |
| Time of stent dilation (s) | 15.0 (13.0-18.0) |
| Balloon dilation pre-stent (n/%) | 91 (31.8) |
| **Medicine used postoperation** |  |
| Aspirin (n/%) | 286 (100.0) |
| Nitrates (n/%) | 274 (95.8) |
| Statins (n/%) | 280 (97.9) |
| β receptor blockers (n/%) | 260 (90.9) |
| ACEIs/ARBs (n/%) | 199 (69.6) |
| Calcium channel blockers (n/%) | 102 (35.7) |

Data were presented as mean value ± standard deviation, count (percentage) or median (25^th^-75^th^ quantiles).

CAD: coronary artery disease; BMI: body mass index; DM: diabetes mellitus; LVEF: left ventricular ejection fraction; MAP: mean arterial pressure; FBG: fasting blood-glucose; Scr: serum creatinine; SUA: serum uric acid; cTnI: cardiac troponin I; NT-proBNP: N-terminal pro brain natriuretic peptide; TG: triglyceride; TC: total cholesterol; LDL-C: low-density lipoprotein cholesterol; HDL-C: high-density lipoprotein cholesterol; Hs-CRP: high-sensitivity C-reactive protein; ESR: erythrocyte sedimentation rate; WBC: white blood cell; LAD: left anterior descending branch; LCX: left circumflex artery; RCA: right coronary artery; PCI: percutaneous coronary intervention; DES, drug-eluting stent; ACEIs/ARBs: angiotensin converting enzymes inhibitors/angiotensin receptor blockers.
