# Supplementary material for: Potential of circulating pro‐angiogenic microRNA expressions as biomarkers for rapid angiographic stenotic progression and restenosis risks in coronary artery disease patients underwent percutaneous coronary intervention
Source: J Clin Lab Anal. 2019 Sep 8;34(1):e23013. doi: 10.1002/jcla.23013 (PMC6977144; doi:10.1002/jcla.23013)
Supplement: Supplementary file 3 [file JCLA-34-e23013-s003.docx]

**SUPPLEMENTARY TABLE** **3** Predictive effect of miRNAs relative expression on RASP by logistic regression analysis

| miRNAs | Logistic regression model | | | |
| --- | --- | --- | --- | --- |
|  | P value | OR | 95%CI | |
|  |  |  | Lower | Higher |
| **Univariate logistic regression** |  |  |  |  |
| let-7b | 0.701 | 1.024 | 0.908 | 1.155 |
| let-7f | **<0.001** | 0.216 | 0.127 | 0.368 |
| miR-17-5p | 0.228 | 0.849 | 0.651 | 1.108 |
| miR-17-3p | 0.383 | 1.128 | 0.861 | 1.477 |
| miR-18a | 0.084 | 0.747 | 0.537 | 1.040 |
| miR-19a | **<0.001** | 0.575 | 0.465 | 0.711 |
| miR-19b-1 | 0.050 | 0.716 | 0.513 | 1.000 |
| miR-20a | 0.208 | 0.851 | 0.662 | 1.094 |
| miR-92a | **0.008** | 0.745 | 0.599 | 0.926 |
| miR-126 | **<0.001** | 0.409 | 0.305 | 0.549 |
| miR-130a | 0.060 | 0.684 | 0.460 | 1.017 |
| miR-210 | **<0.001** | 0.374 | 0.271 | 0.517 |
| miR-296 | **0.001** | 0.613 | 0.462 | 0.812 |
| miR-378 | 0.132 | 0.842 | 0.673 | 1.053 |
| **Multivariate logistic regression with Forward Stepwise (Conditional) method** | | | | |
| let-7f | **<0.001** | 0.293 | 0.155 | 0.554 |
| miR-19a | **<0.001** | 0.571 | 0.435 | 0.750 |
| miR-126 | **<0.001** | 0.433 | 0.306 | 0.614 |
| miR-210 | **<0.001** | 0.404 | 0.271 | 0.604 |
| miR-296 | **0.027** | 0.645 | 0.437 | 0.952 |

Predictive effect of miRNAs relative expression on RASP were determined by univariate and multivariate logistic regression analyses with Forward Stepwise (Conditional) method. P value <0.05 was considered significant (in bold).

RASP: rapid angiographic stenotic progression; OR: odds ratio; CI: confidence interval.
